# miRNA-Driven Regulation of Endothelial-to-Mesenchymal Transition Differs among Thoracic Aortic Aneurysms

**DOI:** 10.3390/cells13151252

**Published:** 2024-07-25

**Authors:** Sonia Terriaca, Maria Giovanna Scioli, Fabio Bertoldo, Calogera Pisano, Paolo Nardi, Carmela Rita Balistreri, Daniele Magro, Beatrice Belmonte, Luca Savino, Amedeo Ferlosio, Augusto Orlandi

**Affiliations:** 1 Anatomic Pathology, Fondazione Policlinico Tor Vergata, 00133 Rome, Italy; terriacasonia093@gmail.com (S.T.); luca.savino@ptvonline.it (L.S.); 2 Anatomic Pathology, Department of Biomedicine and Prevention, Tor Vergata University, 00133 Rome, Italy; ferlosio@med.uniroma2.it (A.F.); orlandi@uniroma2.it (A.O.); 3 Cardiac Surgery Unit, Department of Surgery, Tor Vergata University, 00133 Rome, Italy; fabio.bertoldo@ptvonline.it (F.B.); lindapisano82@gmail.com (C.P.); pa.nardi4@libero.it (P.N.); 4 Cellular and Molecular Laboratory, Department of Biomedicine, Neuroscience and Advanced Diagnostics (Bi.N.D.), University of Palermo, 90134 Palermo, Italy; carmelarita.balistreri@unipa.it (C.R.B.); daniele.magro2497@gmail.com (D.M.); 5 Tumor Immunology Unit, Department of Health Sciences, University of Palermo, 90134 Palermo, Italy; beatrice.belmonte@unipa.it; 6 Azienda sanitaria Provinciale di Catania (ASP), 95124 Catania, Italy; 7 Department of Biomedical Sciences, Catholic University Our Lady of Good Counsel, 1001 Tirana, Albania

**Keywords:** thoracic aortic aneurysms, Marfan syndrome, bicuspid aortic valve, endothelial cells, endothelial-to-mesenchymal transition, tissue and circulating miRNAs, miR-632, miR-126-5p

## Abstract

Thoracic aortic aneurysms (TAAs) represent a serious health concern, as they are associated with early aortic dissection and rupture. TAA formation is triggered by genetic conditions, in particular Marfan syndrome (MFS) and bicuspid aortic valve (BAV). During the aneurysmatic process, aortic endothelial cells can undergo endothelial-to-mesenchymal transition (End–MT) with consequent phenotypic and functional alterations. We previously documented that MFS TAA is characterized by miR-632–driven End–MT exacerbation, whereas in BAV aortopathy, the occurrence of this process remains still controversial. We investigated the End–MT process and the underlined regulatory mechanisms in BAV, TAV and MFS TAA tissues. Gene expression and immunohistochemical analysis were performed in order to analyze some important miRNAs and genes characterizing End–MT. We documented that BAV endothelium maintains the expression of the endothelial homeostasis markers, such as *ERG*, *CD31* and miR-126-5p, while it shows lower levels of miR-632 and mesenchymal markers compared with MFS. Interestingly, we also found higher levels of miR-632 in MFS patients’ blood. Our findings definitively demonstrate that the End–MT process does not characterize BAV that, among the other TAAs, better maintains the endothelial features. In addition, our results suggest miR-632 as a promising diagnostic/prognostic factor in MFS aortopathy.

## 1. Introduction

Marfan syndrome (MFS) is a genetic condition associated with the risk of Thoracic Aortic Aneurysm (TAA) development [1]. MFS is a connective tissue disorder caused by mutations in the FBN1 gene, which codes for the extracellular glycoprotein fibrillin-1 that lead to Transforming Growth Factor β (TGF-β) signaling hyper-activation, vascular wall weakness and TAA onset at an early age [2,3]. The bicuspid aortic valve (BAV) is the most common congenital heart valve defect. In addition to valve dysfunction, BAV is associated with TAA development. The BAV condition is associated with different genetic mutations in *NOTCH1*, *TGFβR2*, *FBN1*, *SMAD6*, *GATA5* and *GATA6* genes [4,5,6].

VSMCs have been considered the most important cell type in TAA pathogenesis, but also endothelial cells play a critical role in it [7]. Some studies reported that endothelial cells can undergo endothelial-to-mesenchymal transition (End–MT), a process in which cells switch from an endothelial to a mesenchymal phenotype, losing cell-to-cell contact and cell polarity [8,9]. End–MT, a specific form of epithelial-to mesenchymal-transition (EMT) originally discovered in endocardium during cardiac development [10], shares many similarities with EMT and is activated during similar biological processes and diseases [11,12]. End–MT can also be induced in response to injury such us oxidative stress, inflammation, or hemodynamic changes [13]. It has been demonstrated that the pathways associated with the End–MT activation are TGF-β, NOTCH and Wnt/β Catenin signaling [8,9,14]. Moreover, the End–MT process seems to be regulated also by miRNAs. miRNAs are small non-coding RNAs that regulate gene expression post-transcriptionally [15]. It has been reported that miRNA deregulation is associated with the End–MT process [9,16]. An miRNA directly involved in End–MT activation in MFS is reported to be miR-632 [9]. Our previous work reported that miR-632 up-regulation in MFS TAA inhibited the DnaJ heat shock protein family (Hsp40) member B6 (DNAJB6) [9]. We demonstrated that the down-regulation of DNAJB6 in MFS TAA tissues led to Wnt/β Catenin activation associated with End–MT exacerbation [9]. Moreover, we proved that miR-632 up-regulation was induced by TGF-β hyper-activation [9]. It is known that after TGF-β1 binds to TβRII/TβRI complexes, it initiates canonical and non-canonical signaling [17]. The canonical TGF-β signaling pathway uses Smad2 and/ or Smad3 to transfer signals, while the non-canonical TGF-β signaling pathway involves ERK/MAPK kinase and N-κB [18]. However, it still remains to be clarified whether the canonical or non-canonical TGF-β pathway or both is activated in MFS.

On the other hand, miR-126-5p has been negatively associated with End–MT, as its role is reported to be to maintain vascular integrity and favor endothelial homeostasis [19]. miR-126-5p is reported to be under the control of the endothelial transcription factor ERG, encoded by an ETS-related gene, that transactivates the miR-126-5p promoter region [20,21,22]. In addition, TRAF-6 and PIK3R2 have been proved to be gene targets of miR-126-5p [23,24]. The recent findings regarding the End–MT process in BAV are still controversial. Some studies reported that BAV endothelium is characterized by homeostasis maintaining its physiological function compared with TAV (Tricuspid Aortic valve) TAA [20]. Moreover, it has been reported that aortic endothelial cells from patients with aortic aneurysm and bicuspid aortic valve have down-regulation of Notch signaling [25]. The latter is reported to induce the End–MT in response to its stimulation by different Notch ligands [26] so it has been demonstrated that its dysregulation in BAV aortic endothelial cells fail to activate Notch-dependent endothelial-to-mesenchymal transition [25]. On the other hand, other studies demonstrated that BAV showed End–MT and discussed the possible contribution of that phenomenon to TAA development in BAV patients. In particular, Gould et al. reported that ROBO4 mutation in BAV endothelial cell results in impaired barrier function and activation of the End–Mt process [27]. The study of Maleky et al. also evidenced the molecular signature of an End–MT/EMT transition-like process, associated with instability of intimal cell junctions and activation of the RHOA pathway in the intima and media layers of ascending aorta in BAV patients [28]. In this work, we want to definitively clarify the presence or absence of the End–MT process in BAV and its regulation by differentially expressed miRNAs, comparing BAV, TAV and MFS TAAs aortic and serum samples.

## 2. Materials and Methods

### 2.1. Tissue and Serum Sample Collection

In this study, we analyzed aortic tissue samples deriving from TAV, BAV and MFS patients (n = 30 each group,) undergoing elective surgical procedures for aortic replacement, collected between 2020 and 2024 in the Department of Cardiosurgery of Tor Vergata University of Rome. In addition, 10 mL of whole peripheral blood was withdrawn from some of those enrolled patients and healthy donors (3 patients for each conditions) and collected into EDTA tubes before surgery. MFS diagnosis was made according to the clinical criteria defined by Ghent’s nosology (see Supplemental Table S1 for clinical characteristics) [29] (See Supplemental Table S1). The diagnosis of Marfan syndrome was also confirmed by genetic analysis of FBN1 mutations by using Ion S5 Next-Generation Sequencing (Thermo Fisher Scientific, Waltham, MA, USA). Successively, all variant findings of FBN1 were validated using Sanger Sequencing. All the MFS patients displayed missense mutations involving cysteine residues (cysteine substitutions) in which the expression of fibrillin is not reduced, but its functionality is altered (see Supplemental Table S1). Clinicians have also collected demographic and clinical features of all patients including medical histories pertinent to aortic disease and co-morbidity conditions (see Supplemental Table S1). The exclusion criteria used during the TAV, BAV and MFS patient enrollment were: coronary artery diseases, endocarditis, aortic dissection, kidney failure, liver disease, tumors, autoimmune diseases, diabetes, infections, etc. (see Supplemental Table S1). BAV, TAV and MFS patients enrolled had the mean ages of 77, 58 and 36 years, respectively. The ascending aorta and aortic root diameter were evaluated by transthoracic echocardiography in preoperatively and in the operating room (Supplemental Table S1); diameter measurements were also performed by using helical computed tomography image analysis techniques. The diameter of aortic annulus, the sinuses of Valsalva, and the proximal ascending aorta (above 2.5 cm of the sinotubular junction) in the parasternal long-axis view as well as the dimensions of the aortic arch from the suprasternal view were measured by transesophageal echocardiography [30] (Supplemental Table S1). The surgical procedures for ascending aorta replacement or for the button Bentall operation were previously reported [31]. The study was approved by the local ethics committee (Protocol no. 179/18-01-Aorta-2018 and Protocol “PNRR-MR1-2022-12376699”), and all patients signed the informed consent form.

### 2.2. Immunohistochemical Analysis

Excised aortic tissue samples, derived from patients who underwent surgery for aortic replacement (n = 30 samples for each condition, totalizing 90 patients), were used for immunohistochemical analysis. In detail, serial 4 µm thick paraffin sections from 10% neutral-buffered formalin-fixed aortic tissue samples were incubated with the following primary antibodies: mouse mono-clonal anti-CD31 (1:50, Dako, Santa Clara, CA, USA), anti-vimentin (1:200, Santa Cruz Biotechnology, Santa Cruz, CA, USA), anti-NF-κΒ p65 (1:100, Santa Cruz Biotechnology), rabbit monoclonal anti-phosphoSMAD3 (1:200; Abcam, Cambridge, UK) and rabbit poly-clonal anti-pTGF-βR1 (1:50, Invitrogen Waltham, MA, USA). Positive and negative controls were used, as reported [32]. Immunohistochemical evaluation was performed by calculating the percentage of positive cells/field (along the entire section, at 20× magnification) and reported as the mean ± SEM (standard error of mean) of the total fields.

### 2.3. Gene Expression Analysis on Tissue Samples

We performed gene expression analysis using RNA and miRNA extracted from endothelium of residual aortic tissue samples derived from MFS, TAV and BAV patients who underwent surgery. In particular, we used frozen aortic tissue samples derived from 9 patients (3 patients for each condition) belonging to the cohort of 90 patients. Before proceeding with RNA extraction, aortic adventitia and tunica media were removed. Endothelium was scraped, and the TRI Reagent^®^ (Sigma Aldrich, St. Louis, MO, USA) was added for total RNA and miRNA extraction according to the manufacturer’s protocols, as already reported [33]. We evaluated the quality of RNA used for gene expression analysis in two steps. Firstly, we quantified the RNA extracted by UV absorption using a spectrophotometer (EZDROP 1000 Micro-Volume spectrophotometer, Resnova, Roma, Italy). In particular, we evaluated the RNA yield and its absorbance at 260/280 and 260/230. For all analysis, we used pure RNA with an OD (optical density) A260/A280 and OD A260/A230 equal to 2.0 or more. Successively, the integrity of RNA was evaluated with running it on 15% denaturing acrylamide gel [34]. A MystiCq microRNA cDNA Synthesis Mix kit (Sigma Aldrich, St. Louis, MO, USA) or the SuperScript III (Invitrogen, Thermo Fisher Scientific, Waltham, MA, USA) were used for reverse-transcribed 700 ng of total RNA. Real-time PCR analysis was performed using SYBR Green (BioRad, Hercules, CA, USA) and specific primers (Supplemental Table S2). The expression of U6 was used to normalize hsa-miR-632 and has-miR126-5p expression, while the other genes were normalized to the expression of GAPDH (Supplemental Table S2). Relative gene expression analyses were calculated using the comparative ΔΔCT method (Supplemental Table S3), as reported [35]. Fold change was considered significant for values > 2.0 and <0.5. The analysis was performed in triplicate. In order to reduce the intra- and inter-variability, we performed the assay on three different pooled samples (3 patients for each condition, totaling 9 patients).

### 2.4. Gene Expression Analysis on Serum Samples

Once collected, blood samples derived from healthy donors (Controls), TAV, BAV and MFS patients and collected before surgery (3 patients for each condition, belonging to the cohort of 90 patients) were immediately centrifuged at 1300× *g* for 10 min at RT to obtain serum and frozen at −80  °C. RNA extraction was performed by adding 1 mL of TRI Reagent^®^ (Sigma Aldrich) for every 200 µL of serum and 1.5 µL of cel-miR-39 spice-in-kit (Norgen Biotek, Thorold, Canada), according to the manufacturer’s protocols. We evaluated the quality of RNA as reported above. Successively, 400 ng of RNA was reverse-transcribed using the specific miRNA reverse transcription kit (Norgen Biotek). We used MystiCq microRNA Primer HSA-miR-632 (Merck KGaA, Darmstadt, Germany), MystiCq microRNA Primer HSA-miR-126-5p (Merck KGaA), cel-miR-39 Forward PCR Primer (Norgen Biotek) and Universal PCR Reverse Primer (Norgen Biotek) to analyze the expression of circulating miRNAs. Changes in gene expression levels were calculated using the comparative ΔΔCT method (see Supplemental Table S4) [35]. miRNAs were normalized to the expression of cel-miR-39, as reported [36]. Fold change was considered significant for values > 2.0 and <0.5. The analysis was performed in triplicate. The assay was carried out on 3 patients for each condition.

### 2.5. Statistical Analysis

In this study, we analyzed only normally distributed and continuous variables between three independent experimental groups (TAV, BAV and MFS). Data were reported as mean ± SEM (standard error of mean), and differences between groups were analyzed by using the unpaired *t*-test, considering statistically significant *p* values < 0.05. Statistical analyses were performed using SPSS software (IBM, SPSS Statistics, Chicago, IL, USA. version 23).

## 3. Results

### 3.1. Different miRNA Deregulation Characterizes the Endothelium of Thoracic Aortic Aneurysms

**Figure 1:**
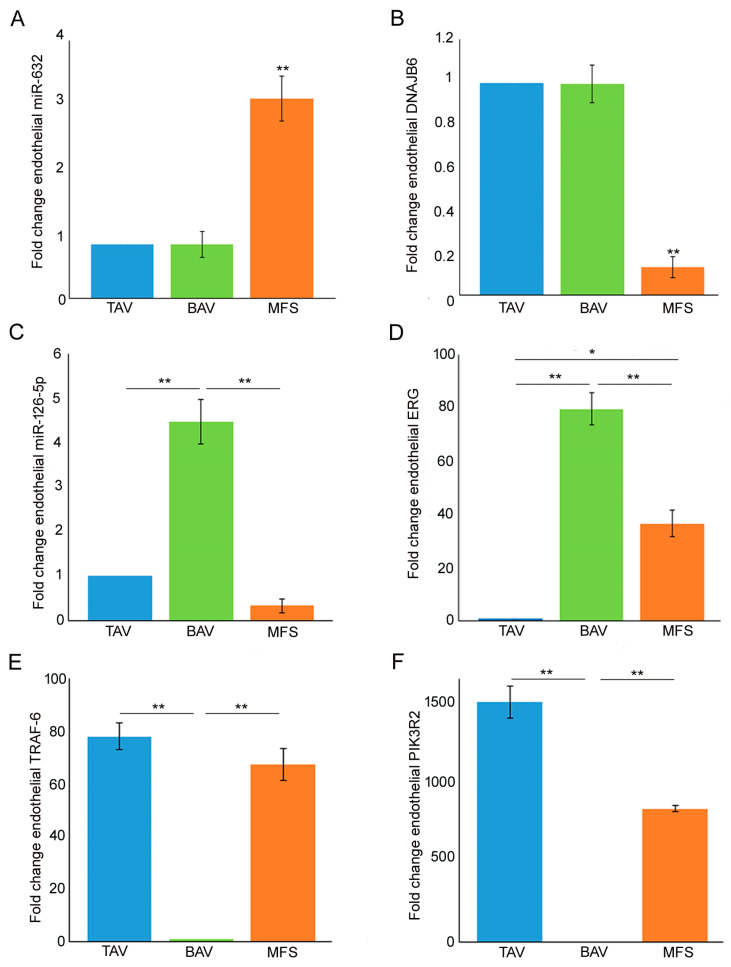
MFS, BAV and TAV aortic endothelia are characterized by differently expressed miRNAs. (**A**) Gene expression analysis shows a greater miR-632 up-regulation in the aortic endothelium of MFS patients compared with TAV and BAV (TAV group is used as calibrator). (**B**) Gene expression analysis of DNAJB6 document its strong down-regulation in MFS endothelium compared with TAV and BAV (TAV group is used as calibrator). (**C**) Gene expression analysis of miR-126-5p displays its up-regulation in BAV compared with TAV and MFS endothelium. (**D**) Gene expression analysis displays a significant *ERG* down-regulation in TAV and MFS endothelium (TAV group is used as calibrator). (**E**,**F**) Gene expression analysis of TRAF-6 and PIK3R2 evidenced their strong down-regulation in BAV compared with TAV and MFS aortic endothelium (BAV group is used as calibrator). The analyses were performed in triplicate on three different pooled samples (3 patients for each pool, totaling 9 patients. Unpaired *t*-test: * and ** indicate *p* < 0.05 and *p* < 0.01, respectively.

In order to evaluate miR-632 and miR-126-5p expression in the different types of TAAs, we performed gene expression analysis on endothelium of BAV, TAV and MFS aortic samples. In our previous work [9], we evidenced the strong miR-632 up-regulation and DNAJB6 down-regulation associated with exacerbation of the End–MT process, comparing aortic endothelium of MFS and TAV patients. In this work, we found the same deregulation also comparing with the BAV endothelium (Figure 1A *p* < 0.01 for MFS vs. TAV and MFS vs. BAV; Figure 1B *p* < 0.01 for MFS vs. TAV and MFS vs. BAV). Successively, we analyzed miR-126-5p in the same aortic samples, discovering its up-regulation in BAV endothelium (Figure 1C, *p* < 0.01 for BAV vs. TAV and BAV vs. MFS). We previously demonstrated the association between the transcription factor ERG and miR-126-5p, as ERG transactivates the miR-126-5p promoter region, and their up-regulation in BAV compared with TAV aortas [20]. In this work, we examined ERG expression in BAV, TAV and MFS aortic endothelia. Our results demonstrated that ERG expression is higher in BAV (with the highest miR-126-5p levels) but down-regulated in MFS and TAV endothelia (Figure 1D; *p* < 0.01 for BAV vs. TAV and BAV vs. MFS and *p* < 0.05 for MFS vs. TAV). Moreover, we also investigated the expression of *TRAF-6* and *PIK3R2*, reported to be the gene targets of miR-126-5p [23,24]. Our results showed the down-regulation of those genes in BAV patients that expressed the highest levels of miR-126-5p (Figure 1E *p* < 0.01 for BAV vs. TAV and BAV vs. MFS; Figure 1F; *p* < 0.01 for BAV vs. TAV and BAV vs. MFS).

### 3.2. End–MT Is Exacerbated in MFS Endothelium Compared with TAV and BAV

**Figure 2:**
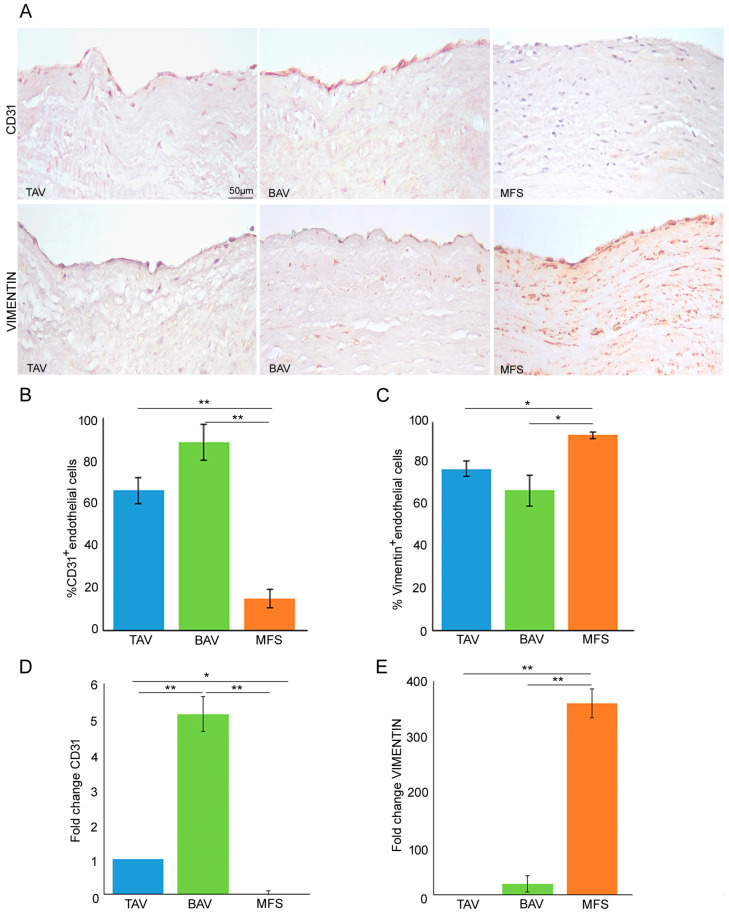
BAV is not characterized by End–Mt but preserves endothelial phenotype. (**A**) Representative images and (**B**,**C**) semiquantitative evaluations of CD31 and vimentin immunostainings display a high percentage of CD31^+^ endothelial cells as well as a reduced percentage of vimentin^+^ endothelial cells in BAV and TAV compared with MFS endothelium. The results are reported as the mean ± SEM. Scale bar = 50 µm. (**D**) Gene expression analysis shows a significant *CD31* up-regulation in BAV compared with TAV and MFS aortic endothelia (TAV group is used as calibrator). (**E**) Gene expression analysis displays *VIMENTIN* up-regulation in MFS compared with TAV and BAV. Immunohistochemical analysis was performed on MFS (n = 30), TAV (n = 30) and BAV (n = 30) aortic samples. Gene expression analyses were carried out in triplicate on three different pooled samples (3 patients for each pool, totaling 9 patients). Unpaired *t*-test: * and ** indicate *p* < 0.05 and *p* < 0.01, respectively.

In order to definitively clarify the presence or not of the End–MT process in BAV samples, we analyzed the expression of endothelial and mesenchymal markers (CD31 and vimentin), performing gene expression and immunohistochemical analysis on BAV, TAV and MFS aortas. Immunohistochemical analysis displayed an increased CD31 expression as well as decreased vimentin levels in BAV and TAV compared with MFS endothelium (Figure 2A,B *p* < 0.01 for MFS vs. BAV and MFS vs. TAV; Figure 2A,C *p* < 0.05 for MFS vs. BAV and MFS vs. TAV). Gene expression analysis showed a *CD31* up-regulation only in BAV endothelium compared with those of TAV and MFS as well as *VIMENTIN* up-regulation in MFS endothelium compared with BAV and TAV (Figure 2D *p* < 0.01 for BAV vs. TAV, MFS vs. BAV and *p* < 0.05 MFS and TAV; Figure 2E *p* < 0.01 for MFS vs. TAV and MFS vs. BAV). Among those types of TAAs, MFS evidenced the exacerbation of the End–MT process. Those findings definitively clarify that BAV aorta, as well as that of TAV, is not characterized by the End–MT process but preserves endothelial phenotype.

### 3.3. Non-Canonical TGF-β Signaling Is Hyper-Activated in MFS Aorta

**Figure 3:**
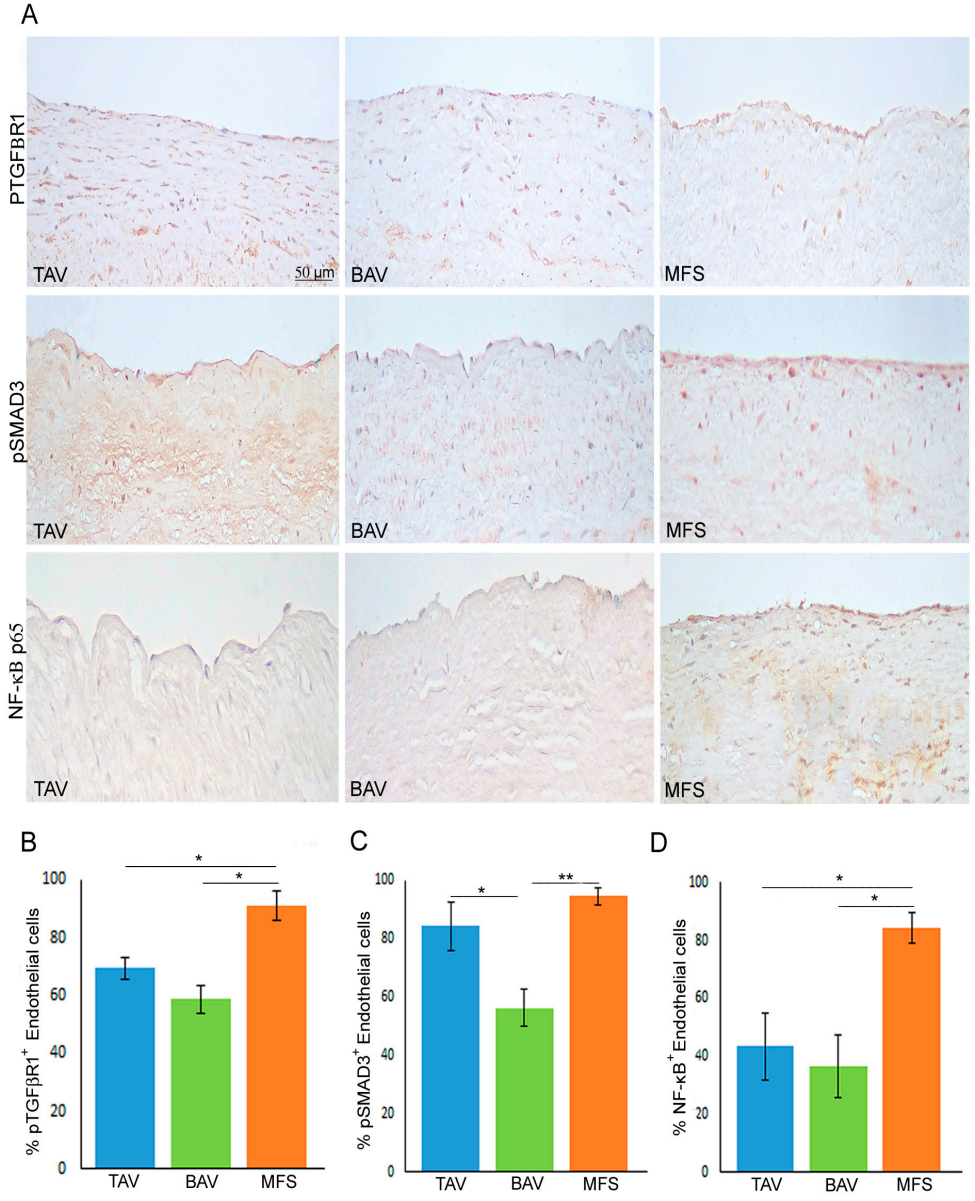
Non-canonical TGF-β signaling is hyper-activated in MFS aorta. (**A**) Representative images and (**B**–**D**) semiquantitative evaluations of pTGFβR1, pSMAD3 and NF-κB p65 immunostainings show the high percentage of pTGFβR1^+^ and NF-κB^+^ endothelial cells in MFS endothelium compared with TAV and BAV as well as the high percentage of pSMAD3^+^ endothelial cells in MFS and TAV endothelia (no differences) compared with BAV. The results are reported as the mean ± SEM. Immunohistochemical analysis was performed on MFS (n = 30), TAV (n = 30) and BAV (n = 30) aortic samples. Scale bar = 50 µm. Unpaired *t*-test: * and ** indicate *p* < 0.05 and *p* < 0.01, respectively.

As reported above, MFS is characterized by TGF-β1 hyper-activation associated with miR-632 deregulation and the End–MT process [9]. Therefore, we analyzed pTGFβR1 expression in BAV, TAV and MFS aortas through immunohistochemical analysis, evidencing that MFS endothelium showed the highest expression of that protein compared with BAV and TAV aortas (Figure 3A,B, *p* < 0.05 for MFS vs. TAV and MFS vs. BAV). In order to understand which of the two TGF-β signaling pathways (canonical and non-canonical) is activated in MFS, we analyzed the expression of pSMAD3 as a protein of canonical TGF-β signaling and NF-κB as a protein of non-canonical TGF-β pathway. Immunohistochemical analysis evidenced that pSMAD3 has a higher expression both in MFS and TAV and a reduced expression in BAV (Figure 3A,C, *p* < 0.05 for TAV vs. BAV and *p* < 0.01 for MFS vs. BAV), suggesting that canonical TGF-β signaling is active both in TAV and MFS. On the other hand, NF-κβ expression was very high in MFS but not in BAV and TAV (Figure 3A,D, *p* < 0.05 for MFS vs. TAV and MFS vs. BAV). Those findings suggest that TGF-β hyper-activation in MFS leads to activation both of canonical and non-canonical TGF-β pathways that could be responsible for the miR-632 up-regulation with the consequent End–MT exacerbation.

### 3.4. MFS Patients’ Serum Showed the Same miR-632 Deregulation Observed in Aortic Endothelium

**Figure 4:**
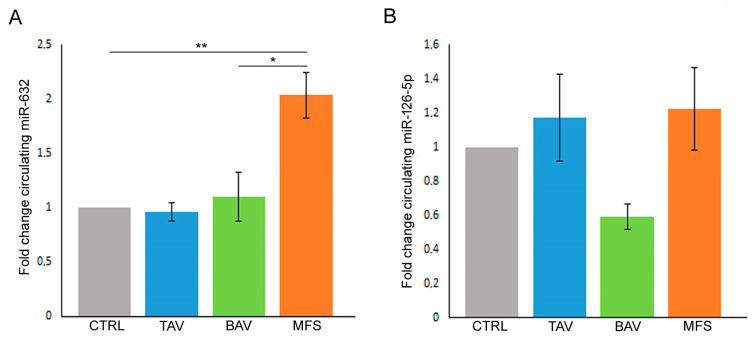
MFS patients’ serum showed the same miR-632 deregulation observed in aortic endothelium. (**A**) Gene expression analysis displays the miR-632 up-regulation in the pre-operatory serum of MFS patients compared with the serum of TAV, BAV and control (CTRL) subjects (CTRL group is used as calibrator). (**B**) Gene expression analysis shows that the circulating levels of miR-126-5p do not reflect the results obtained in aortic tissue samples (CTRL group is used as calibrator). The results are reported as the mean ± SEM. Gene expression analyses were carried out in triplicate on three patients/subjects for each conditions. We reported the Unpaired *t*-test: * and ** indicate *p* < 0.05 and *p* < 0.01, respectively.

In order to verify if miR-632 and miR-126-5p deregulations, related to the End–MT process and observed in aortic tissues, are also present in blood samples, we performed gene expression analysis on serum samples derived from controls (healthy donors), TAV, BAV and MFS patients collected before surgery. Our results showed for the first time that miR-632 up-regulation, observed in MFS aortic tissues, is also present in serum samples of MFS patients compared with controls, TAV and BAV (Figure 4A; *p* < 0.01 for MFS vs. CTRL and TAV and *p* < 0.05 for MFS vs. BAV), further confirming that miR-632 deregulation is MFS-specific. On the other hand, results regarding the circulating levels of miR-126-5p did not reflect those obtained in aortic tissue samples, probably the circulating levels of that miRNA are influenced by other factors (Figure 4B).

## 4. Discussion

Our results evidence that MFS and BAV aortic endothelia are characterized by miRNA differential expression associated with their different pathogenetic mechanisms. In particular, we observed that MFS endothelium displays a strong miR-632 up-regulation and DNAJB6 inhibition compared with BAV and TAV. In our previous work, we have already demonstrated that miR-632 up-regulation induces, by DNAJB6 down-regulation and Wnt/β Catenin signaling activation, the End–MT exacerbation in MFS comparing with TAV aortic endothelium [9]. The present study confirms our previous data, evidencing that the increased tissue and circulating levels of miR-632 is an MFS-specific feature. In addition, we demonstrated that MFS shows a significant reduction in miR-126-5p compared with TAV and BAV. On the other hand, BAV aortic endothelium displays a conserved expression of this homeostatic miRNA as well as a high expression of its activator ERG compared with TAV and MFS. However, TAV endothelium showed strong ERG down-regulation compared with MFS and BAV; this is likely due to vascular aging, since TAV patients are older than BAV and MFS patients, and ERG function is reported to be decreased in aging [37]. As concerns the different ERG expression between BAV and MFS, the latter are the youngest patients but show a lower ERG expression than BAV (older than MFS). Therefore, ERG expression is associated with aging (difference between TAV and MFS as well as TAV and BAV) but also to the endothelial homeostasis, which is altered in the pathogenetic mechanisms of MFS (difference between BAV and MFS).

In addition, the highest miR-126-5p expression, among TAAs, associates with a significant down-regulation of its targets, *TRAF-6* and *PIK3R2*.

As reported above, those miRNAs and gene targets are related to endothelial functions that are lost with the End–MT process. As previously mentioned, in the literature there are conflicting results regarding the End–MT process in BAV aortopathy [25,27,28]. In order to definitively clarify the occurrence of the End–MT process in BAV, we analyzed the expression of endothelial and mesenchymal markers in BAV, TAV and MFS endothelia. Our results confirm that MFS is characterized by an exacerbation of the End–MT process compared with BAV that maintained the endothelial phenotype as well as a physiological homeostasis (miR-126-5p retain). In our results, the expression of endothelial/mesenchymal markers, analyzed by immunohistochemical analysis, is not statistically significant between TAV and BAV, unlike gene expression of the same markers; this is likely due to the different sensitivity of the method.

In our previous work, we proved that the miR-632-mediated End–MT process in MFS is induced by TGF-β signaling hyper-activation [9], but without clarifying which TGF-β pathway, the canonical (Smad3) and/or non-canonical (ERK, NF-κB) was activated. As we expected, pTGFβR1 is strongly expressed in MFS compared with BAV and TAV endothelia. Moreover, we found that pSMAD3 is highly expressed both in MFS and TAV (no significant differences) and reduced in BAV. Instead, the subunit NF-κB p65 is strongly expressed in MFS compared with TAV and BAV. Altogether, those results highlight that the pathogenic mechanisms observed in MFS endothelium are likely related in part to the hyper-activation of the non-canonical TGF-β pathway.

In addition, as reported above, we investigated miRNA deregulation also in patients’ serum. Interestingly, we found miR-632 up-regulation also in MFS patients’ serum, evidencing the strong disease specificity of the miRNA. As regards miR-126-5p, data obtained in patients’ serum do not reflect the results obtained in aortic tissue, indicating that other factors affect the levels of circulating miR-126-5p.

In conclusion, our findings definitively demonstrate that BAV aorta as well as TAV aorta are not characterized from an evident End–MT compared with MFS, in which this process is highly exacerbated. Moreover, unlike miR-126-5p that is not disease-specific, the strong correlation between the miR-632, both in tissues and blood, suggest it as a potentially promising prognostic/therapeutic target in the clinical management of MFS patients. However, given the limited number of samples analyzed, dealing with rare genetic diseases, further studies on a larger cohort of patients is needed in order to achieve our goal.

## Data Availability

Data are contained within the article or in Supplementary Table S1.

## Supplementary Materials

The following supporting information can be downloaded at: https://www.mdpi.com/article/10.3390/cells13151252/s1, Table S1: Demographic and Clinical characteristic of the Study Population; Table S2: Sequences of primers used for Real-time PCR; Table S3: Data obtained from Real time PCR of aortic tissue samples derived from TAV, BAV and MFS patients; Table S4: Data obtained from Real time PCR of blood samples derived from TAV, BAV and Marfan patients.
