# Supplementary material for: miRNA-Driven Regulation of Endothelial-to-Mesenchymal Transition Differs among Thoracic Aortic Aneurysms
Source: Cells. 2024 Jul 25;13(15):1252. doi: 10.3390/cells13151252 (PMC11312012; doi:10.3390/cells13151252)
Supplement: Supplementary file 1 [file cells-13-01252-s001.zip › cells-3059685-supplementary.pdf]

Supplemental Table S1. Demographic and Clinical characteristic of the Study Population.

Demographic and Clinical Characteristics	BAV TAA (n=30)	TAV TAA (n=30)	MARFAN TAA (n=30)
Age (years) ± SD	58.2 ± 9.7	77.8 ± 5.6	35.2 ± 15.3
Male sex	22 (73%)	16 (53%)	15 (50%)
Race	Caucasian	Caucasian	Caucasian
Smoking	7 (23%)	6 (20%)	7 (23.3%)
Hypertension	6 (20%)	6 (20%)	3 (10%)
Diabetes	0	0	0
Renal Failure	0	0	0
Tumors	0	0	0
Coronary artery diseases	0	0	0
Liver disease	0	0	0
Infections	0	0	0
Endocarditis	0	0	0
Aortic dissection	0	0	0
Autoimmune disease	0	0	0
Type of aortic valve	BAV	TAV	TAV
FBN1 mutation	0	0	YES (100% missense mutation in Cys)
Highly arched palate	0	0	13 (43.3%)
Thumb sign	0	0	3 (10%)
Positive wrist	0	0	6 (20%)
Severe miopia	0	0	7 (23%)
Scoliosis	0	0	7 (23%)
Skin striae	0	0	3 (10%)
Mitral Valve Prolapse	0	0	7 (23%)
Dilated aorta diameter ± SD	54.2 ± 3.4 (ascending)	59.45 ± 7.7 (ascending)	51.8 ± 4.0 (root)
Coronary Ostia Dislocation	7 (23%)	6 (20%)	N.A
Left Ventricular/Aortic Valve disjunction	3 (10%)	3 (10%)	N.A
Asymmetric Dilation of Ascending Aorta	3 (10%)	5 (16.7%)	NA
Valsalva Sinuses Prolapse	3 (10%)	8 (26.7%)	N.A

Abbreviations: BAV, Bicuspid Aortic Valve; TAA, Thoracic aortic aneurysm; TAV, Tricuspid Aortic Valve; N.A., Not Available (Data); SD, Standard Deviation.

Supplemental Table S2. Sequences of primers used for Real-time PCR

GENE/miRNA	PRIMER SEQUENCES/Cat. Number
MystiCq microRNA hsa-miR-632	Forward: 5’-GUGUCUGCUUCCUGUGGGA-3’
MystiCq microRNA hsa-miR-126-5p	Forward: 5’CAUUAUUACUUUUGGUACGCG-3’
MystiCq microRNA RNU6-1 (for endothelial miRNAs)	MIRCP00001 (Merk KGaA)
cel-miR-39 Forward PCR Primer (for circulating miRNAs)	microRNA (cel-miR-39) Spike-In Kit Product Cat. N° 59000 (Norgen Biotek)
MystiCq® Universal PCR Primer	MIRUP (Merk KGaA)
Universal PCR Reverse Primer	Cat. N° 54410 (Norgen Biotek)
h-DNAJB6	Forward:5’-CATGCCTCACCCGAGGATATT-3’<br>Reverse: 5’-CCTCCGCTACTTGCTTGAATTT-3’
h-ERG	Forward: 5′-GGAGTGGGCGGTGAAAGA-3′<br>Reverse: 5′-AAGGATGTCGGCGTTGTAGC-3
h-CD31	Forward: 5’-GCCAGGTTGAGAAACTCTGC-3’<br>Reverse: 5’-TGGGTTGTACCTTCCAGGAG-3’
h-VIMENTIN	Forward: 5’-TCCAAGTTTGCTGACCTC-3’<br>Reverse: 5’-CAGTGGACTCCTGCTTTG-3’
h-TRAF-6	Forward: 5’-GTTGCTGAAATCGAAGCACA-3’<br>Reverse: 5’-CGGGTTTGCCAGTGTAGAAT-3’
h-PIK3R2	Forward: 5’-GCACCACGAGGAACGCACTT-3’<br>Reverse: 5’-CGTCCACTACCACGGAGCAG-3’
h-GAPDH	Forward: 5’-ACGGATTTGGTCGTATTGG-3’<br>Reverse: 5’-GATTTTGGAGGGATCTCGC-3’

Supplemental Table S3. Data obtained from Real time PCR of aortic tissue samples derived from TAV, BAV and MFS patients.

Gene	Delta CT value TAV (pool)	Delta CT value BAV (pool)	Delta CT value MARFAN (pool)	Delta Delta CT (vs TAV or BAV as Relative Calibrators)	Fold change($2^{-\Delta\Delta CT}$)
Endothelial miR-632 (normalized to U6 expression)	7,16	7,07	5,53	Marfan vs TAV(Calibrator)= -1,63	Marfan vs TAV(Calibrator)=3,09
				BAV vs TAV (Calibrator)=-0,08	BAV vs TAV (Calibrator)=1,05
DNAJB6 (normalized to GAPDH expression)	-0,50	-0,43	2,39	Marfan vs TAV(Calibrator)= 2,90	Marfan vs TAV(Calibrator)=0,13
				BAV vs TAV (Calibrator)= 0,07	BAV vs TAV (Calibrator)= 0,94
Endothelial miR-126-5p (normalized to U6 expression)	5,16	2,99	6,78	Marfan vs TAV(Calibrator)= 1,62	Marfan vs TAV(Calibrator)=0,32
				BAV vs TAV (Calibrator)= - 2,16	BAV vs TAV (Calibrator)= 4,48
ERG (normalized to GAPDH expression)	7,74	1,43	2,55	Marfan vs TAV(Calibrator= -5,19	Marfan vs TAV(Calibrator)= 36,64
				BAV vs TAV (Calibrator)= -6,31	BAV vs TAV (Calibrator)= 79,59
TRAF-6 (normalized to GAPDH expression)	0,67	6,96	0,88	Marfan vs BAV(Calibrator= -6,07	Marfan vs BAV(Calibrator)= 67,41
				TAV vs BAV (Calibrator)=-6,29	TAV vs BAV (Calibrator)=78,11
PIK3R2 (normalized to GAPDH expression)	0,09	10,64	0,93	Marfan vs BAV(Calibrator= -9,71	Marfan vs BAV(Calibrator)= 837,59
				TAV vs BAV (Calibrator)= -10,55	TAV vs BAV (Calibrator)=1503,99
CD31 (normalized to GAPDH expression)	4,17	1,82	10,60	Marfan vs TAV(Calibrator)= 6,43	Marfan vs TAV(Calibrator)=0,01
				BAV vs TAV (Calibrator)=-2,36	BAV vs TAV (Calibrator)=5,13
VIMENTIN (normalized to GAPDH expression)	4,85	0,51	-3,63	Marfan vs TAV(Calibrator)= -8,48	Marfan vs TAV(Calibrator)= 357,82
				BAV vs TAV (Calibrator)=-4,34	BAV vs TAV (Calibrator)= 20,28

Supplemental Table S4. Data obtained from Real time PCR of blood samples derived from TAV, BAV and Marfan patients.

Gene	Delta CT values of TAV samples	Delta CT values of BAV samples	Delta CT values of MARFAN samples	Delta CT values CTRLS/healthy donors (Calibrator)	Delta Delta CT (vs CTRL)	Fold change($2^{-\Delta\Delta CT}$)
Circulating miR-632 (normalized to cel-miR-39 expression)	TAV 1: 8,78	BAV 1: 9,05	MARFAN 1: 7,13	CTRL 1: 8,44	Marfan (delta ct mean) vs CTRL (calibrator , delta ct mean):-1,07	Marfan vs CTRL (calibrator): 2,09
	TAV 2: 9,06	BAV 2: 8,72	MARFAN 2: 7,97	CTRL 2:9,05	TAV (delta ct mean)vs CTRL (calibrator, delta ct mean): 0,05	TAV vs CTRL (calibrator): 0,96
	TAV 3: 9,01	BAV 3: 8,51	MARFAN 3: 8,39	CTRL 3: 9,19	BAV (delta ct mean) vs CTRL (calibrator, delta ct mean):-0,13	BAV vs CTRL (calibrator): 1,09
Circulating miR-126-5p (normalized to cel-miR-39 expression)	TAV 1: 9,73	BAV 1: 11,13	MARFAN 1: 10,39	CTRL 1: 8,63	Marfan (delta ct mean) vs CTRL (calibrator, delta ct mean): -0,29	Marfan vs CTRL (calibrator): 1,23
	TAV 2:10,68	BAV 2: 11,25	MARFAN 2: 10,10	CTRL 2: 10,78	TAV (delta ct mean) vs CTRL (calibrator, delta ct mean): -0,22	TAV vs CTRL (calibrator): 1,16
	TAV 3:9,75	BAV 3: 11,01	MARFAN 3:9,43	CTRL 3: 11,41	BAV (delta ct mean)vs CTRL (calibrator, delta ct mean): 0,85	BAV vs CTRL (calibrator): 0,55
